# Extraction and Characterization of Chitin from the Beetle *Holotrichia parallela* Motschulsky

**DOI:** 10.3390/molecules17044604

**Published:** 2012-04-17

**Authors:** Shaofang Liu, Jie Sun, Lina Yu, Chushu Zhang, Jie Bi, Feng Zhu, Mingjing Qu, Chen Jiang, Qingli Yang

**Affiliations:** Shandong Peanut Research Institute, No. 126 Fushan Road, Qingdao 266100, China

**Keywords:** insect, chitin, *Holotrichia parallela*, characterization, extraction

## Abstract

Insect chitin was isolated from adult *Holotrichia parallela* by treatment with 1 M HCl and 1 M NaOH, following by 1% potassium permanganate solution for decolorization. The yield of chitin from this species is 15%. This insect chitin was compared with the commercial α-chitin from shrimp, by infrared spectroscopy, X-ray diffraction, scanning electron microscopy, and elemental analysis. Both chitins exhibited similar chemical structures and physicochemical properties. Adult *H. parallela* is thus a promising alternative source of chitin.

## 1. Introduction

Chitin, found in arthropods (insects, crustaceans, arachnids and myriapods), is the second most abundant biopolymer after cellulose [1]. Structurally, it is a derivative of cellulose with only an acetamido group replacement at position C-2, *i.e*., β-(1,4)-2-acetamido-2-deoxy-D-glucose. Chitin is classified into three types according to the different orientations of its microfibrils: α-chitin (anti-parallel chains), β-chitin (parallel chains), and γ-chitin (the combination of parallel and anti-parallel chains) [1,2]. Chitin is insoluble in most solvents because of its compact structure. Therefore, chemical modifications of chitin are performed to obtain more soluble analogs, among which, chitosan, derived by partial *N*-deacetylation of chitin, is the most common such derivative.

Chitin and chitosan are attracting great interest because of their beneficial biological properties, such as biodegradability, biocompatibility, non-antigenicity and non-toxicity [3,4]. Since they are versatile biopolymers, their potential applications in various industrial fields are being actively investigated. For example, chitin and chitosan have been documented to be useful as antimicrobial, emulsifying, thickening and stabilizing agents in the food industry [5]. They have also shown notable bioactivity in biomedical fields, including wound healing promotion, immune system enhancement, and hemostatic, hypolipidemic and antimicrobial activity [6,7].

Traditionally, chitin is prepared mainly from crab and shrimp shells obtained as byproducts in the seafood industry. Chitin is also a primary component in insect cuticles. Therefore, insects are an alternative source of chitin and, consequently, of chitosan. Recently, the production of chitin and chitosan from insect sources has drawn increased attention. First, insects possess enormous biodiversity and represent 95% of the animal kingdom [8]. Therefore, they offer a tremendous potential as a natural resource for chitin and chitosan production. Furthermore, insect cuticles have lower levels of inorganic material compared to crustacean shells, which makes their demineralization treatment more convenient [9]. Until now, however, only limited numbers of insect species have been documented to be sources of chitin [9,10,11,12,13].

In this study, the isolation and characterization of chitin from adult *Holotrichia parallela* Motschulsky is described. *H. parallela* is a beetle species belonging to the Melolonthidae subfamily of the Scarabaeoidea family. These insects are field crop pests in China. In addition, they have also been used as food and traditional medicinal herbs in China and East Asia [14,15,16]. Every year, large amounts of *H. parallela* adults are captured for the control of this pest in fields. Our institute is investigating the possibility of rearing *H. parallela* [17]. This will provide a source for studies on the identification of active components from this beetle, including chitin and its derivative chitosan. Here, chitin was purified from *H. parallela* adults using acid and alkaline treatments, followed by decolorization with potassium permanganate. The physiochemical properties of chitin were characterized by infrared spectroscopy (IR), X-ray diffraction (XRD), elemental analysis and scanning electron microscopy (SEM) methods. These physicochemical properties were also compared with those of shrimp chitin.

## 2. Results and Discussion

### 2.1. Chitin Extraction

Chitin is a major component of the insect cuticle, which is always covalently bound to catechol compounds and sclerotin-like proteins [10]. The most common method for chitin extraction from insects involves two steps, an acidic step to remove catechols and a basic step to remove the cuticle proteins, as had been mentioned elsewhere for insect chitin isolation [9,10]. Generally, the acidic treatment conditions used for extraction from insects are moderate in comparison to crustacean exoskeletons. The reason for this is that insects have low levels of inorganic material (less than 10%) as compared to crustacean shells (20–40%) [18,19].

The ash content of chitin is indicative of the effectiveness of the method used for removal of inorganic materials. From Table 1, the adult *H. parallela* contained 5.5% ash on a dried basis, while the ash content of chitin extracted from this species dropped to 2.2%. This result suggested that the demineralization with 1 M HCl for 30 min was an effective method for decreasing the ash content in adult *H. parallela*. The nitrogen content of chitin from adult *H. parallela* decreased to 6.3%, which is lower than the theoretical value of 6.9% for pure chitin. This also indicated an effective deproteinization process in chitin extraction. In comparison, the commercial chitin used as standard contained 1.6% ash and 6.2% nitrogen.

**Table 1:** Chemical composition of dried raw materials and chitin from adult *H. parallela* and shrimp chitin.

	Moisture (%)	Ash (%)	Nitrogen (%) ^a^
**Dried raw materials**	3.63 ± 0.04	5.53 ± 0.23	11.29 ± 0.03
**Beetle chitin**	7.12 ± 0.16	2.20 ± 0.13	6.33 ± 0.06
**Shrimp chitin**	7.64 ± 0.06	1.59 ± 0.12	6.22 ± 0.06

^a^ measured by the Kjeldahl method.

The yield of chitin from adult *H. parallela* is around 15%. The yields of chitin from other insects varied with species and their development stages. *Bombyx mori* larva cuticle and silkworm pupa exuviae were reported to yield 15–20% of chitin [9]. A higher chitin yield of 36% was reported for cicada sloughs [12]. In comparison, the yields of α-chitin from crustacean shells are about 7–40%, depending on the species [18]. Adult *H. parallela* is thus a promising alternative source of chitin.

### 2.2. Characterization of Chitin

#### 2.2.1. IR Analysis

**Figure 1:**
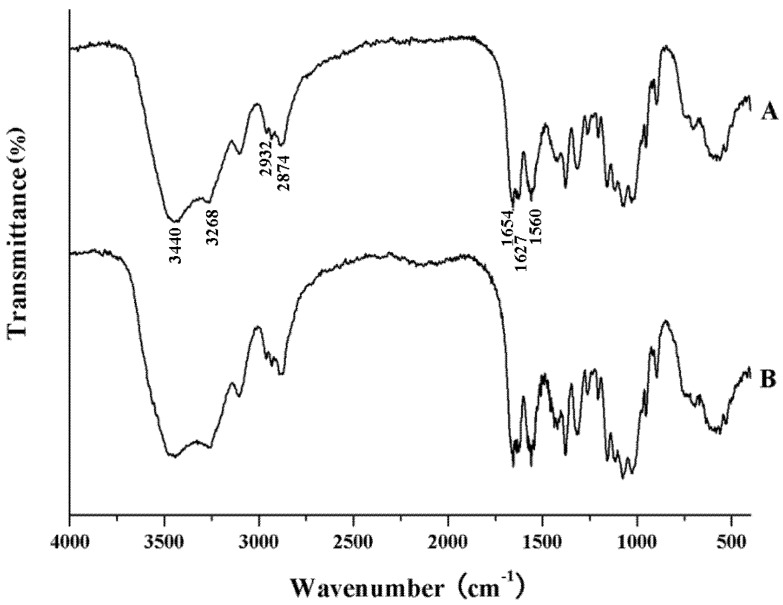
IR spectra of α-chitin from shrimp (**A**) and from adult *H. parallela* (**B**).

The IR spectra of chitin from shrimp and *H. parallela* are shown in Figure 1. They were quite similar, and comparable to those of α-chitin from other sources in the literature [10,20]. The spectra were characterized by three significant amide bands at 1,654, 1,560 and 1,310 cm^−1^, which correspond to the amide Ι stretching of C=O, the amide ΙΙ of N-H and amide ΙΙΙ of C-N, respectively. It is important to note that the amide Ι band of α-chitin splits at 1,654 and 1,627 cm^−1^, which is attributed to the two types of H-bonds formed by amide groups in the antiparallel alignment present in crystalline regions of α-chitin [21,22]. Table 2 showed the assignment of the relevant bands of chitins from shrimp and *H. parallela*. From this result, it was demonstrated that both chitins are in α form. The degree of acetylation (DA) values of chitin from shrimp and *H. parallela* calculated using the formula (1) were 94.3% and 93.1%, respectively.

**Table 2:** Assignments of the relevant bands of IR spectra of chitin from shrimp and adult *H. parallela*.

Assignments	Wave Number (cm^−1^)	
	Beetle Chitin	Shrimp Chitin
ν (O-H)	3,424	3,440
ν (N-H)	3,262	3,268
ν (COCH_3_)	2,934	2,932
ν (C-H)	2,884	2,874
ν (C-O)	1,655	1,654
ν (C=O of *N*-acetyl group)	1,624	1,627
δ (N-H of *N*-acetyl group)	1,560	1,560

#### 2.2.2. Elemental Analysis

Elemental analysis of chitins from shrimp and *H. parallela*, including the carbon, nitrogen and hydrogen contents and C/N ratio are shown in Table 3. From chitin, the nitrogen contents of the sample come mainly from protein and chitin. The total nitrogen content of the beetle before chitin extraction was ~11% (by Kjeldahl method). After extraction, the resultant nitrogen content dropped to 6.45%. This value was lower than the theoretical value (6.9%) calculated for a completely acetylated chitin, indicating the minimum amount of protein left.

**Table 3:** The elemental analysis of chitin from shrimp and *H. parallela* and the corresponding degree of acetylation (DA).

Samples	Content (%)			C/N
	N	C	H	
Shrimp chitin	6.24	43.75	6.40	7.07
Insect chitin	6.45	44.36	5.92	6.88

#### 2.2.3. XRD Analysis

**Figure 2:**
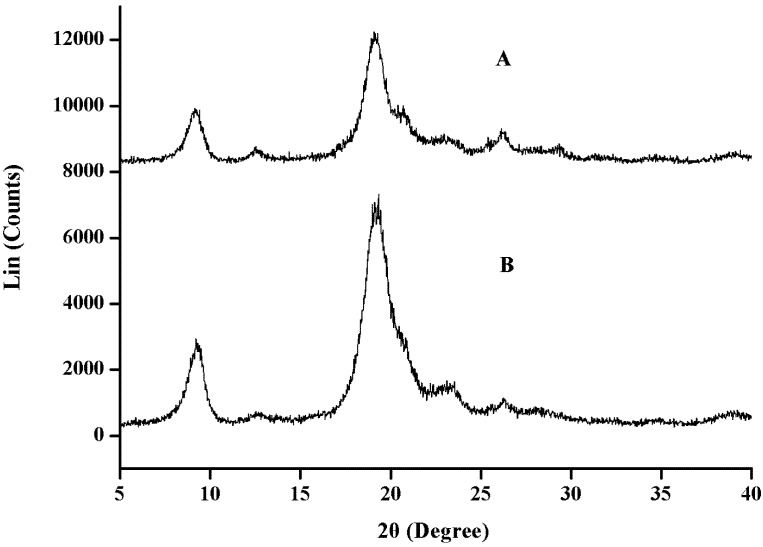
The XRD patterns of chitin from shrimp (**A**) and *H. parallela* (**B**).

Figure 2 represents the XRD pattern for α-chitin from shrimp and *H. parallela*. From the results, both chitin samples showed similar XRD patterns, with strong reflections at 9.2 and 19.1° and minor reflections at 12.6, 22.9 and 26.2° (Figure 2A,B). Chitin from other beetle species like cicada sloughs was reported to show a similar result, with reflections at 2θ 9.20, 12.60, 19.18, 20.68, 23.30 and 26.48° [12]. The crystalline index (CrI) of chitin was calculated from the XRD data. The results showed that both chitins have nearly the same crystallinity, 89.17 and 89.05% for chitin from shrimp and *H. parallela*, respectively. Chitin from cicada sloughs had a similar crystallinity of 89.7% (CrI_110_). However, a much lower crystallinity, only 54 and 58%, was found in chitins from larva cuticles and silkworm pupa exuviae (*Bombyx mori*), respectively [9]. The authors inferred that the low molecular weight compound catechol that remained in the insect chitin was probably the reason for the low crystallinity.

#### 2.2.4. Scanning Electron Microscopy (SEM)

**Figure 3:**
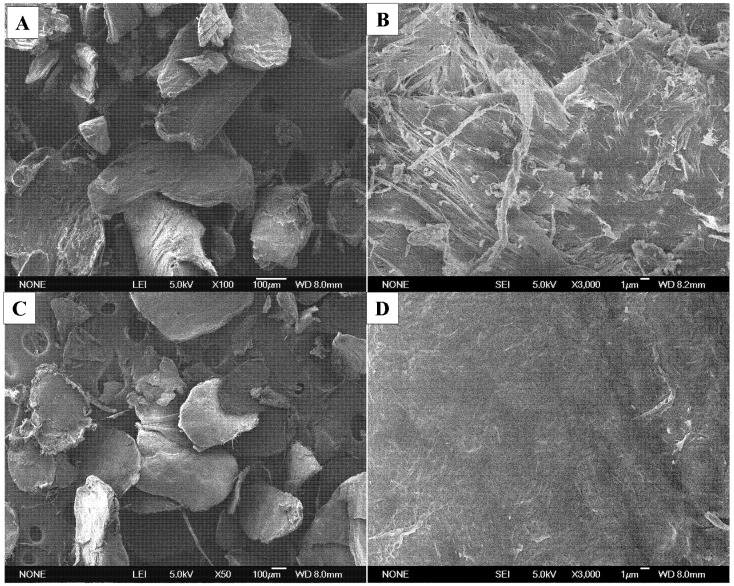
SEM micrographs for α-chitin from shrimp at 50× magnification (**A**) and at 3,000× magnification (**B**) and from adult *H. parallela* at 50× magnification (**C**) and at 3,000× magnification (**D**).

Figure 3 shows the SEM photographs of commercial chitin from shrimp and chitin from *H. parallela*. Both samples exhibited rough and thick surface morphology under electron microscopic examination at 50× magnification.

Similar results can be seen in the SEM photographs of the beetle chitin from cicada sloughs [12]. At higher magnification (3,000×), chitin from shrimp (Figure 3B) was found to be distinctly arranged in a microfibrillar crystalline structure, more noticeable than chitin from adult *H. parallela* (Figure 3D).

## 3. Experimental

### 3.1. Isolation of Chitin from Adult H. parallela

Adult *H. parallela* were captured in July in peanut fields in the suburbs of Qingdao, Shandong Province, China, as part of the usual control of this pest. The beetles were starved for 48 h to eliminate gut contents, washed with water and killed by freezing. They were allowed to thaw at room temperature and then air-dried at 50 °C for 2 days. The dried beetles were milled to a powder to pass through a 20-mesh screen and stored at 4 °C in airtight containers. The powder (5 g) was treated with 1 M HCl solution (250 mL) at 100 °C for 30 min to remove minerals and catechols. The demineralization step was followed by rinsing with distilled water until neutrality was reached. Deproteinization was performed using alkaline treatment with 1 M NaOH (250 mL) solution at 80 °C for 24 h, and the product was washed with distilled water until the pH became neutral. For the purpose of decolorization, the precipitate was treated further with 1% potassium permanganate solution (100 mL) for 1 h. Finally, lightly brown chitin was washed with distilled water and dried at 50 °C in a dry heat sterilizer.

### 3.2. Infrared Spectra (IR) Analysis

Chitin samples were characterized from 4,000 to 400 cm^−1^ by infrared spectrophotometry (WGH-30/30A, Gangdong Technology Ltd., Tianjin, China) in KBr pellets. Commercial chitin from shrimp (Sigma) was used as standard. The DA of both chitin samples was determined by comparing the absorbance of the measured peak to that of the reference peak. The DA was calculated from the absorbance (A) ratios according to the following equation [10]:

      DA = (A_1655_/A_3450_) × 100       (1)

### 3.3. Elemental Analysis

Elemental analysis was performed using a Vario EL III analyzer (Elementar, Hanau, Germany) at the Institute of Chemistry, Qingdao University of Science and Technology according to the standard operation procedures provided by the manufacturer.

### 3.4. X-ray Chitin Powder Diffraction

XRD analysis was used to detect the crystallinity of chitins prepared, and their patterns were recorded using a D/Max-rA diffractometer (Rigaku, Tokyo, Japan) with Cu radiation at the Institute of Chemistry, Qingdao University of Science and Technology. Data were collected at a scan rate of 1°/min with the scan angle from 5° to 40°. The crystalline index (CrI) was determined by the following equation:

      CrI_110_ = [(I_110_ − I_am_)/I_110_] × 100       (2)

where I_110_ is the maximum intensity at 2θ ≌ 20° and I_am_ is the intensity of amorphous diffraction at 2θ ≌ 16°.

### 3.5. Scanning Electron Microscopy (SEM)

The surface morphology of chitin was examined with JSM-6700F scanning electron microscope (JEOL, Tokyo, Japan) at the Institute of Chemistry, Qingdao University of Science and Technology. The dried samples were ground, fixed on an adhesive tape and then coated with a thin gold layer by a sputter coater. The SEM was conducted at 5.0 kV.

### 3.6. Composition Analysis

Moisture and ash were assayed according to the Association of Official Analytical Chemists methods (AOAC, 2006) (AOAC methods 934.01 and 942.05). Nitrogen content was measured by the Kjeldahl method (AOAC method 984.13).

## 4. Conclusions

Chitin was isolated from adult *H. parallela* using standard methods. The low levels of ash and nitrogen contents are indicative of the effectiveness of the chitin extraction method. The characteristics of chitin from adult *H. parallela* were similar to those of commercial chitin from shrimp by IR, XRD, SEM and elemental analysis. The chitin extracted from adult *H. parallela* is thus suitable for chitosan production. Adult *H. parallela* is an alternative source of chitin. The large numbers of *H. parallela* adults captured for the control of this pest in fields every year provide an abundant source for the production of chitin. In addition, attempts to domesticate the beetle will help relieve the impact to ecological systems in the future.
